# Prevalence of COVID-19 Among Typical Ambulatory Care Patients in a District General Hospital in the United Kingdom

**DOI:** 10.7759/cureus.11398

**Published:** 2020-11-09

**Authors:** Muhammad Arif, Mina Maaref Doost

**Affiliations:** 1 Internal Medicine, Watford General Hospital, Watford, GBR

**Keywords:** covid-19, ambulatory care, ppe, pandemic, nosocomial, healthcare workers

## Abstract

Objectives

This observational retrospective study was undertaken to ascertain the prevalence of coronavirus disease 2019 (COVID-19) among typical ambulatory care patients. In our hospital, ambulatory care unit (ACU) was supposed to be a COVID-19 free area, and, hence, as per the guidelines, even basic personal protection equipment (PPE) was not provided during the early phase of pandemic.

Methods

We identified 443 patients who presented to our ACU between March and June 2020 with chest pain or shortness of breath suspected of pulmonary embolism or acute coronary syndrome, which normally makes the bulk of referrals to ACU. As per protocol, patients with COVID-19-like symptoms, e.g., fever, cough, sore throat, and loss of taste and smell, were excluded from ACU. We then, reviewed computed tomography (CT) scans for radiological evidence of COVID-19, and lab data for COVID-19 polymerase chain reaction (PCR) or antibody tests, to find out if any of our patients turned out to be suffering from COVID-19 unexpectedly.

Results

We found 13 patients with radiological or serological evidence of COVID-19, which equates to a prevalence of 2.93% in this cohort of our ambulatory care patients. Four in our patient cohort showed radiological features that were highly suggestive of COVID-19 pneumonia; 47 chest CT scans were performed, which may suggest a prevalence of around 8.5% (4/47) on radiological ground if everyone was offered a CT scan.

Conclusions

Due to limited access to data, our result is likely an underestimation of the actual prevalence of COVID-19 among our ACU patients, highlighting the need to review the safety and PPE guidelines for the ambulatory clinic and any similar out-patient areas.

## Introduction

The coronavirus disease 2019 (COVID-19) is an ongoing worldwide pandemic, at the brink of the second wave, caused by highly contagious, severe acute respiratory syndrome coronavirus 2 (SARS-CoV-2), spreading mainly through airborne droplet route and physical contact with contaminated surfaces. As of October 13, 2020, there have been 37,704,153 confirmed cases of COVID-19 globally, including 1,079,029 deaths, reported to World Health Organization [1], and in the UK, there have been 634,920 confirmed cases, with 43,018 deaths within 28 days of positive test [2].

Considering the contagiousness of COVID-19, the issues related to personal protective equipment (PPE), such as lack of robust guidelines and shortage of supplies, have been major concerns worldwide [3,4]. Healthcare workers (HCW) themselves are particularly at high risk of COVID-19 infection because of more frequent exposure, and they could also contribute to the nosocomial spread as carriers. One study from the UK and the USA estimated that frontline HCW had a 3.4-fold higher risk of getting infected [5].

## Materials and methods

Aims and objectives

In ambulatory care unit (ACU), the medical team deals with patients who are otherwise fit but present with a specific symptom that can be managed as a day case. In ACUs, normally patients and their attendants have to use a shared waiting room and the toilets. Due to limited space and lack of isolation facilities, a policy was put in place in the very beginning of the epidemic to exclude any patients from the ACU with symptoms suggestive of COVID-19, such as fever, cough, and loss of taste or smell (Table 1). Therefore, the members of staff were not required to wear even basic PPE such as masks and gloves. Our ACU accepts referrals of patients with suspected pulmonary embolism (PE) or acute coronary syndrome (ACS) presenting with acute chest pain or shortness of breath (SOB). Referrals to ACU are normally triaged by the Accident & Emergency (A&E) service and then a senior ACU staff and duty consultant. Our aim was to review if any ACU patients in the selected group turn out to be COVID-19 unexpectedly in spite of our exclusion protocol to keep the area and staff safe.

**Table 1:** Inclusion and exclusion criteria CRX, chest X-ray; NEWS, National Early Warning Score; PCR, polymerase chain reaction; PE, pulmonary embolism

Inclusion Criteria	Exclusion Criteria
Acute stable chest pain	Fever
Acute shortness of breath, suspected of PE	Cough
NEWS < 2	Loss of taste or smell
Suspected acute coronary syndrome	Known positive COVID-19 PCR
	Abnormal CXR at the source
	Waiting for COVID-19 swab

Methodology

We retrospectively reviewed our ACU Logbooks for the period between March 3, 2020, and June 15, 2020, and identified patients who presented to ACU with SOB or chest pain suspected of either PE or ACS (Table 2). We reviewed the investigations performed in ACU, including routine blood tests, chest X-ray, computed tomography (CT) scan, CT pulmonary angiogram (CTPA), and hospital lab data on Sunquest Integrated Clinical Environment (ICE) software (Sunquest, Tucson, AZ) to check if any of these patients had COVID-19 polymerase chain reaction (PCR) or COVID-19 antibody tests performed within 30 days following the visit. We did not have access to the community or Public Health England (PHE) data. We based our diagnosis of COVID-19 on either radiological criteria [6,7], PCR, or antibody tests.

**Table 2:** Demographics of the patients A&E, Accident & Emergency; GP, general practitioner

Demographics	
Total patients included	443
Males	219 (49.44%)
Females	224 (50.56%)
Average age (years)	50.51
Referred by A&E service	342 (77.2%)
Referred by GP	86 (19.4%)
Referred by other departments	15 (3.3%)

## Results

**Figure 1:**
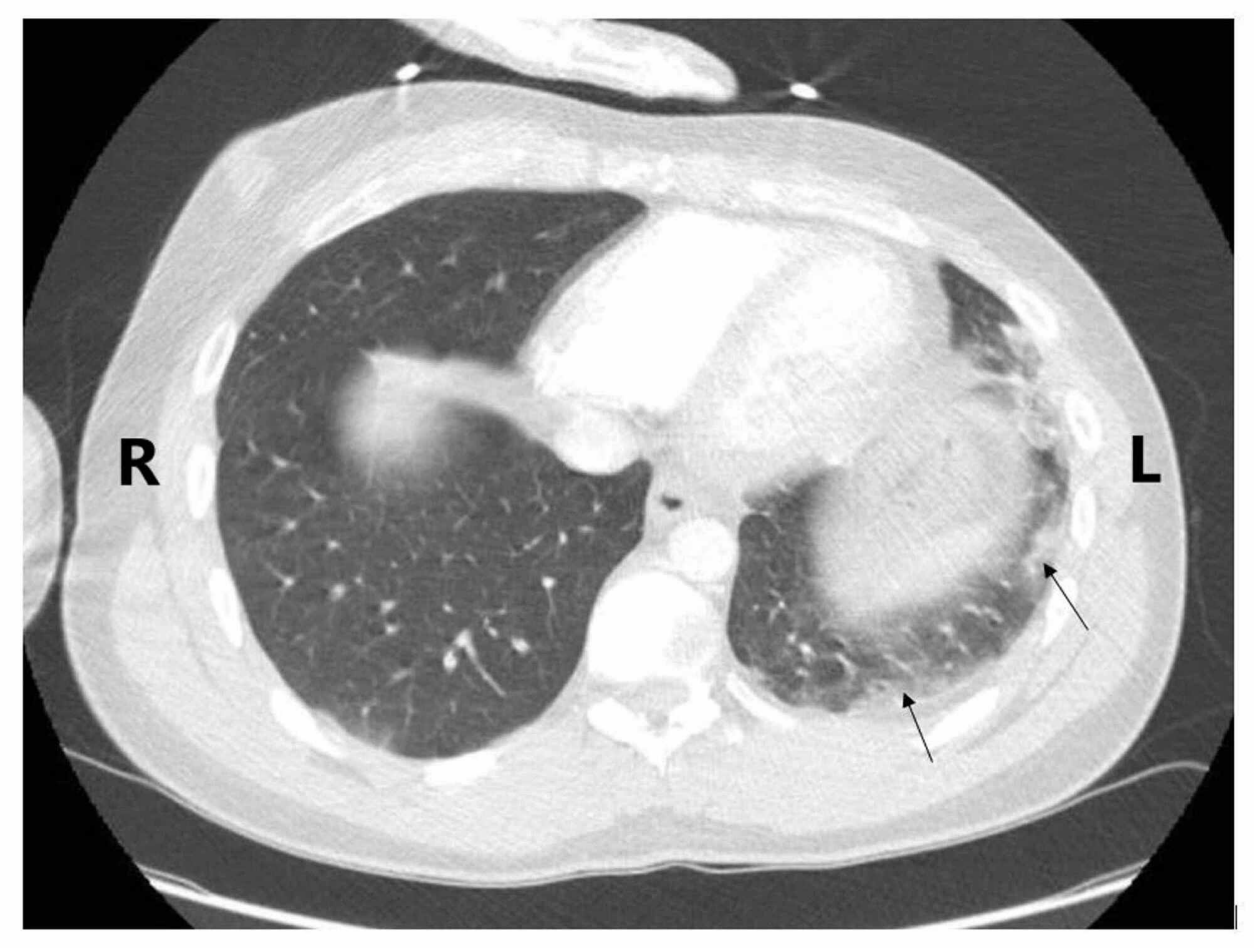
CTPA showing bilateral basal infiltrates, particularly on the left side in this section (see arrows), highly suggestive of COVID-19. CTPA, computed tomography pulmonary angiogram

The total number of patients who fulfilled inclusion criteria was 443 (Table 2). Besides routine blood tests and chest X-ray, majority had troponin and D-dimer tests. A total of 47 patients underwent chest CT scans including 45 CTPA, as per our pathway for PE, and two chest CT for suspicious opacity on the chest X-ray. We applied radiological [6,7] or serological criteria to diagnose COVID-19 and identified 13 patients (Table 3) with evidence of COVID-19, which equates to 2.93% prevalence in our cohort of patients. Four had COVID-19-like changes on the chest CT scans (Figure 1) out of total 47 scans performed, which may indicate a prevalence of around 8.5% (4/47) on radiological ground if everyone was offered a chest CT scan. Two were COVID-19 PCR positive and seven had positive COVID-19 antibody tests. As per our routine, we were not performing COVID-19 PCR or antibody test on any of ambulatory care patients; therefore, these tests must have been performed on a subsequent visit to the hospital. We did not have access to the results of those who might have had their COVID-19 tests conducted in the community.

**Table 3:** Patients with evidence of COVID-19 ACS, acute coronary syndrome; ACU, ambulatory care unit; CT, computed tomography; CTPA, computed tomography pulmonary angiogram; F female; M, male; SOB, PCR, polymerase chain reaction; shortness of breath

	Date of ACU Visit	Age	Sex	Presenting Complaint	Diagnostic Investigation
1.	March 27, 2020	60	M	SOB	COVID-19 PCR positive
2.	April 3, 2020	37	F	Chest pain	COVID-19 antibody detected
3.	April 9, 2020	60	F	Chest pain, dysuria	COVID-19 antibody detected
4.	May 5, 2020	78	M	Chest pain, SOB	CTPA
5.	May 5, 2020	35	M	Chest pain	CTPA
6.	May 8, 2020	29	M	SOB	CTPA
7.	May 12, 2020	57	F	Chest pain	COVID-19 antibody detected
8.	May 21, 2020	79	F	SOB	COVID-19 PCR positive
9.	June 1, 2020	55	F	Tingling in the left arm: ACS?	CT of the thorax/abdomen
10.	June 1, 2020	46	F	Atypical chest pain	COVID-19 antibody detected
11.	June 6, 2020	40	F	Sudden syncope with chest heaviness	COVID-19 antibody detected
12.	June 7, 2020	50	M	Chest pain	COVID-19 antibody detected
13.	June 8, 2020	45	F	Sudden tachycardia: ACS?	COVID-19 antibody detected

## Discussion

This study demonstrates the risk of COVID-19, even in what was supposed to be a COVID-19 free area like ACU, in spite of a well-considerate exclusion protocol in place. The unpredictable nature of this highly contagious disease is only becoming more evident with time and experience, but this exposes frontline HCW to an unacceptable risk [5] while on duty and beyond.

Normally in ACU, the patients and their attendants stay for three to four hours during which they are first enrolled by the reception, triaged by a nurse, seen by a junior doctor, wait for their results in a shared waiting room, and then seen by a consultant. Therefore, during a typical visit, each patient may come in close contact with at least five members of staff from reception to the final consultation, in addition to the other patients.

Our study revealed a prevalence of nearly 3% of COVID-19 in this selected cohort of patients, and possibly up to 8.5% prevalence based on radiological diagnosis on chest CT scans. However, neither we were able to perform COVID-19 test on every ambulatory patient and nor we had access to any external data to check COVID-19 status of all those patients who visited ACU during the study period. Therefore we believe that the actual prevalence could potentially be much higher than shown by our results.

Testing of all patients attending ACU for COVID-19 is not practical due to time and space constraints. Secondly, many studies have demonstrated poor sensitivity of the COVID-19 PCR tests [8]. In hospitals, allocating beds on the basis of PCR test results have proved to be misleading, making management of this crisis even more complex while assuring safety of the hospital staff. Seroprevalence studies have raised concerns about failure to detect people with mild or subclinical COVID-19 disease [9].

Frontline HCWs are particularly at high risk of acquiring COVID-19 infection [5]. A meta-analysis found that the overall proportion of HCW who tested positive for SARS-Co-2 was 11% [10], but another study based on surveillance data from 15 European countries including the UK reported it to be 23.2% from a total of 124,796 reported cases [11]. Majority of HCW reported contact in the healthcare setting (55% in the USA) [12].

HCWs may play a role in asymptomatic transmission of COVID-19 among patients and in family clusters. A cross-sectional study of nearly 2,800 HCWs found that 5.4% of those asymptomatic staff working in COVID-facing areas tested positive compared with 0.6% working in COVID-free wards [13]. However, some studies have reported lower illness severity in HCWs and identified PPE use as the main factor [14].

The reproductive number Ro (the number of people who can acquire infection from an infected person) has been reported to be 2.2-3.3 [15], and it decreases significantly when social distancing measures are in place [16].

In ambulatory care settings, it may be prudent to treat all patients as a potential infection risk given the time constraints and the lack of space. We can keep patients and staff safe by performing heightened screening and by following the appropriate PPE measures for preventing nosocomial spread of COVID-19 [17]. The recently deployed Track and Trace system might help as a screening tool in identifying potentially infected patients when they are referred to the ACU.

Limitations

The limitations of this study were that it was retrospective analysis of patients and that it was based on hospital data only. Not all ACU patients were being tested for COVID-19 as routine, and there was no access to PHE data.

Key learning

1. COVID-19 carriers may be asymptomatic or present with atypical symptoms; therefore, even non-COVID-19 facing areas in the hospitals cannot be considered absolutely safe.

2. Each frontline HCW-patient interaction should be considered as a potential infection risk.

3. PPE guidelines should be reviewed in light of our findings and followed appropriately in the ACUs and out-patient clinics.

## Conclusions

Our study has clearly demonstrated that it is not possible to keep an area like ACU absolutely COVID-free, as we are gradually getting more aware of the atypical nature of the COVID-19. Therefore, it is highly recommended that each patient-staff interaction is regarded as potential risk of infection and necessary PPE protocol must be followed, including masks, gloves, eye covering, and disposable aprons. Hand washing with soap and the use of hand sanitizer is paramount after each patient interaction to keep staff safe and control the spread of COVID-19 infection. As and when rapid point-of-care testing for COVID-19 becomes available, it will be very useful in screening patients before being accepted for ACU, out-patient clinics, and emergency surgery.
